# Asylum seekers’ and refugees’ experiences of accessing health care: a qualitative study

**DOI:** 10.3399/BJGPO.2021.0059

**Published:** 2021-10-13

**Authors:** Ashrafunnesa Khanom, Wdad Alanazy, Lauren Couzens, Bridie Angela Evans, Lucy Fagan, Rebecca Fogarty, Ann John, Talha Khan, Mark Rhys Kingston, Samuel Moyo, Alison Porter, Melody Rhydderch, Gillian Richardson, Grace Rungua, Ian Russell, Helen Snooks

**Affiliations:** 1 Research Fellow, Medical School, Swansea University, Swansea, UK; 2 Lecturer, Midwifery, Majmaah University, Al Majma'ah, Saudi Arabia; 3 Senior Project Manager, Public Health Wales, Policy and International Health WHO Collaborating Centre on Investment for Health and Well-being, Cardiff, UK; 4 Research Officer, Medical School, Swansea University, Swansea, UK; 5 Speciality Registrar in Public Health, Imperial College Healthcare NHS Trust, London, UK; 6 Senior Project Manager, Public Health Wales , Policy and International Health WHO Collaborating Centre on Investment for Health and Well-being, Cardiff, UK; 7 Professor in Public Health,, Medical School, Swansea University, Swansea, UK; 8 Medical Student, School of Medicine, University College Cork, Cork, Ireland; 9 Research Officer, Medical School, Swansea University, Swansea, UK; 10 Public Member and Asylum Seeker, Patient and Public Involvement Members, Swansea University, Swansea, UK; 11 Associate Professor, Swansea University, Swansea, UK; 12 Senior Project Manager and Lead Specialist Advisor, Behavioural Insights, Natural Resources Wales, Cardiff, UK; 13 Senior Professional Advisor to Chief Medical Officer for Wales, Welsh Government, Population Healthcare Directorate, Cardiff, UK; 14 Professor Emeritus (Medicine), Medical School, Swansea University, Swansea, UK; 15 Professor in Health Services Research, Swansea University, Swansea, UK

**Keywords:** asylum seekers, refugees, general practice, delivery of health care, mental health, language

## Abstract

**Background:**

Asylum seekers and refugees (ASRs) often experience poor health in host countries. The United Nations High Commissioner for Refugees (UNHCR) requires hosts to ensure these sanctuary seekers have access to basic health care.

**Aim:**

To identify barriers and facilitators that affect access to health care by ASRs in Wales.

**Design & setting:**

Participatory research approach using qualitative focus groups across Wales, which hosts 10 000 refugees.

**Method:**

Eight focus groups were undertaken with ASRs, support workers, and volunteers (*n* = 57).

**Results:**

Specialist NHS-funded services and grant-aided non-governmental organisations (NGOs) facilitated access to health care, including primary care. Most ASRs understood the role of general practice in providing and coordinating care, but were unaware of out-of-hours services. Reported barriers included: language difficulties, health literacy, unrecognised needs, and the cost of travel to appointments. Participants recognised the importance of mental health, but were disappointed by the state of mental health care. Some feared seeking support for mental health from their GP, and few were aware they had the right to move practice if they were unhappy. Written information about health care was not as accessible to refugees as to asylum seekers (ASs). While some participants read such material before consulting, others struggled to access information when in need. Few participants were aware of health prevention services. Even when they knew about services, such as smoking cessation, these services’ difficulty in accommodating ASRs was a barrier.

**Conclusion:**

The main barriers identified were: availability of interpreters; knowledge about entitlements; and access to specialist services.

## How this fit in

ASRs often experience poor health in host countries as they navigate unfamiliar health systems, often with limited language proficiency.^1–3^ This qualitative research explores their healthcare needs, barriers to access, and facilitators. Support from specialist NHS-funded services and volunteers facilitate access to health care. Remaining challenges include knowledge about entitlements, availability of professional interpreters, and access to specialist and preventive services.

## Introduction

Minimum standards set by the UNHCR requires host nations to ensure ASRs^4,5^ (see Box 1) have access to basic health care.^6,7^ Persecution and the journey to sanctuary affect ASRs’ health. Many have experienced trauma and present with mental illness, infections, and chronic diseases.^8–10^ Improved access to health care can prevent the spread of infection and deterioration in health,^11,12^ and reduce the costly burden on secondary care.^13^ Wales has a long history of welcoming those fleeing persecution. Since 2001, the UK Home Office dispersal policy has brought ASs to Wales, and the Syrian Vulnerable Persons Resettlement Scheme^14^ has been relocating refugees to Wales since 2015. It is estimated that there are some 10 000 refugees in Wales,^15^ which is about 0.3% of the population; in 2019 the National Asylum Support Service (NASS) was supporting approximately 2626 ASs in Wales.^16^ On arrival in Wales, ASs undergo health assessment by specialist AS nurses, including vaccination and tuberculosis screening. Most receive NASS^17^ accommodation in one of four dispersal areas in Wales: Cardiff, Newport, Swansea, and Wrexham. Local AS nurses then register ASs with general practices and signpost them to other services, including dentists. Refugees who arrive in Wales through a national settlement programme^14^ rely on support from the third sector, which can access short-term funding from the Home Office.

Box 1 **Definition of terms ‘asylum seeker’ and ‘refugee’**

An asylum seeker is someone who makes a request to the authorities in the country where they have arrived to seek protection (asylum) under the UN Refugee Convention 1951^4,5^ and are waiting to receive or appeal a decision on their claim for refugee status. Asylum seekers’ movement and right to work are restricted; they receive limited financial support from the state.^15^ Once a claim for asylum is approved, a person is granted refugee status and can access a wider range of public funds, choose where they live, and seek employment. A refugee is someone who has been recognised under the 1951 Refugee Convention as a refugee. In the UK, refugees, asylum seekers, and refused asylum seekers are entitled to receive care free of charge in the same way as any other patient.

ASRs’ experiences and entitlements are shaped by the UK’s rights-based approach to health care,^18^ and access to health care remains free to most ASRs. Nevertheless, barriers arise from ignorance around entitlements,^19–21^ fear of having to pay for services,^20,21^ perceived discrimination,^2,3^ fear of deportation,^3^ and difficulty accessing interpreters.^20^ Challenging appointment systems^3,22^ and restricted access to specialist care lead some to seek emergency care, or avoid care altogether.^1,23^

To the authors’ knowledge no qualitative study has been conducted with recent and long-term ASRs living in Wales about their experiences of health care (primary, community, and secondary care). The Welsh Government has ambitions to create a more equal and healthier Wales,^24^ and the country is seeking to become the world’s first Nation of Sanctuary.^14^ To this end, Public Health Wales commissioned a study^15^ to identify the barriers and facilitators that affect the ability of ASRs to access health care in Wales, aiming to inform new policy interventions that can improve the health and wellbeing of ASRs.

## Method

### Study design

Participatory research was adopted through co-production^25^ as the overarching study design. Eight focus groups were conducted with ASRs, support workers, and volunteers to explore their experiences of ASRs’ accessing health care.^26,27^ The focus groups formed part of a larger mixed-methods study (reported elsewhere),^28^ including a survey, delivered with the involvement of peer researchers and Patient and Public Involvement (PPI) representatives from the ASR community. They informed the research questions, recruitment methods, study information, and focus group interview schedule (see supplementary material). They also validated study findings in light of their understanding and experiences of health care at a stakeholder event attended by health professionals, researchers, and Public Health Wales, and presented findings at the Health Services Research conference.

### Conceptual framework

**Figure 1.**
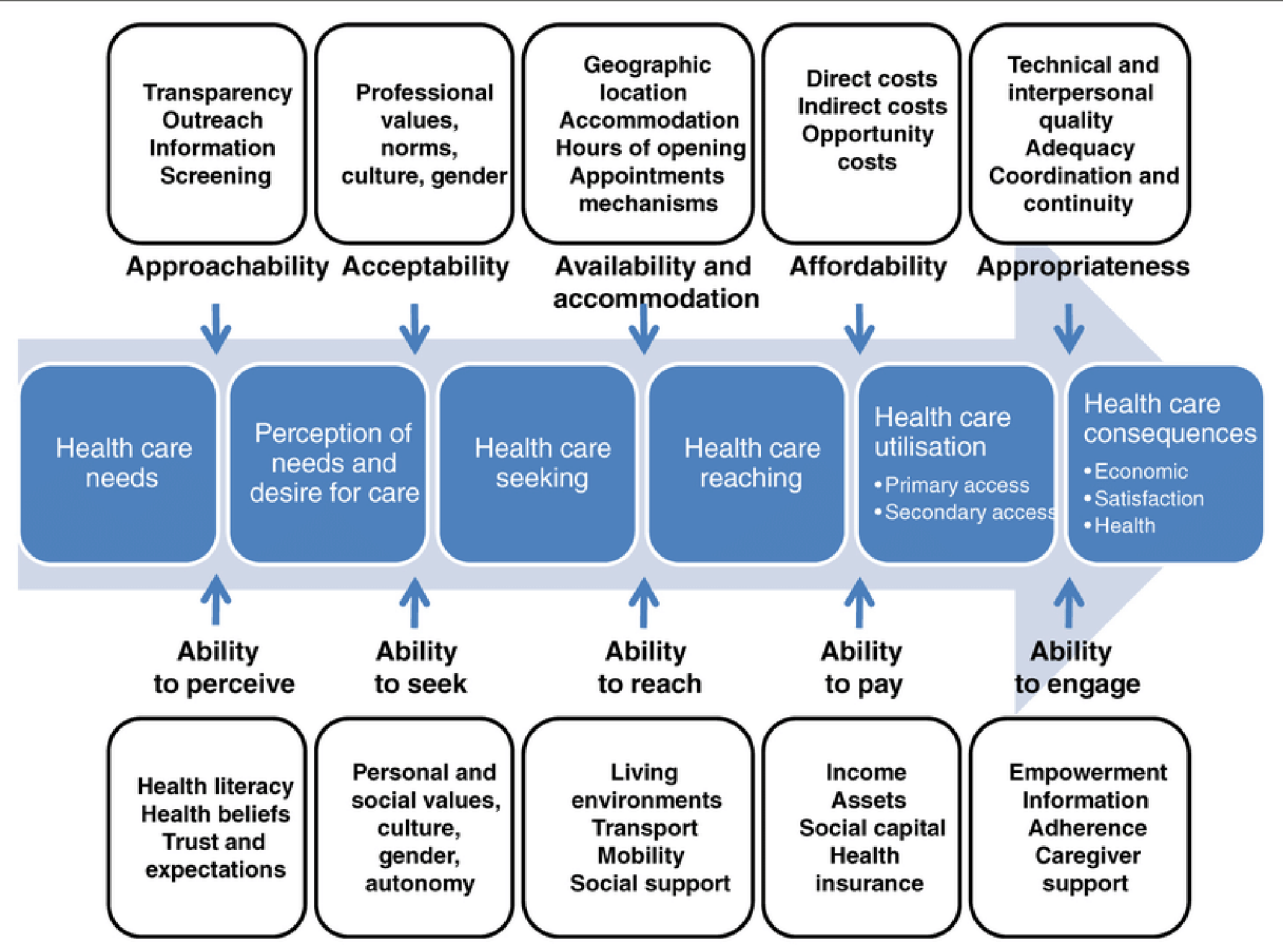
Levesque *et al*’s^29^ conceptual framework of access to health care

No existing theoretical models of access to health care that feature immigration policy or legal status were identified. This study drew on the model of Levesque *et al* ^29^ (Figure 1), which identifies five dimensions of service accessibility: approachability, acceptability, availability, affordability, and appropriateness; and five dimensions of patients’ ability to access services: ability to perceive, ability to seek, ability to reach, ability to pay, and ability to engage. This framework was used to interpret the qualitative data about ASRs’ access to health care.

### Setting

Focus groups were conducted across the four main urban dispersal areas, one semi-urban area, and two rural areas.

### Recruitment and sampling

Convenience sampling was used to recruit participants to the study. GRIPP2 guidelines^30^ were followed for lay involvement in research, and the research team recruited two PPI representatives and four development officers from relevant NGOs — British Red Cross, Displaced People In Action, Ethnic Youth Support Team, and Welsh Refugee Council — to engage the study population. ASRs completing the survey were invited to join focus groups. The initial aim was to include only ASRs; in some instances, however, participants requested that their support workers or volunteers be present to facilitate participation. The volunteers and support workers were able to clarify certain issues raised by the participants in relation to their access to services.

### Data collection

The authors developed a semi-structured focus group topic guide, which was devised and piloted with peer researchers (see supplementary appendix). Health-seeking scenarios were used to encourage participants to discuss general health issues. One experienced qualitative researcher (AK) led all eight focus groups, with two researchers (TK, WA) taking written notes and helping with interpretation. Written consent was obtained from all participants before the focus groups. Questions were asked in English and, when necessary, questions were translated and responses interpreted from: Kurdish (through a professionally trained interpreter); Arabic (through co-author WA, who is a PhD nursing student and native speaker); Bengali; Punjabi; Urdu (through co-authors TK, who is a medical student, and AK, who was a qualified professional interpreter); and Tigrinya (through an asylum seeker who is a native speaker). With participants’ consent, the discussions were recorded and transcribed in English.

### Analysis

Framework analysis,^31,32^ informed by Levesque *et al*’s model,^29^ was used and facilitated by NVivo qualitative data analysis software (version 12). Two of the researchers (AK, BAE) independently read transcripts to identify and refine themes. Discrepancies were resolved in interpretation by discussion and unresolved discrepancies were referred to an experienced qualitative researcher (AP). Peer researchers and PPI members were involved in the review of themes. PPI members were co-authors and were involved in the write up of the study.

## Results

The eight focus groups across Wales had a total of 57 participants; 50 were ASRs (see Table 1) and seven were ASR support workers and volunteers. To overcome cultural barriers, two groups were female only, and two male only. Twenty-four people identified as ASs and 26 as refugees. One person identified as a ‘destitute asylum seeker’ having exhausted all appeal rights. Thirteen people had been living in the UK for more than 5 years with one person still seeking asylum for themselves and their family for 14 years. A high proportion of study participants were able to communicate in English, reflecting their length of stay in the UK. Focus groups included a minimum of three ASRs and one support worker, and a maximum of eight ASRs and two support workers. The focus groups lasted between 45 and 80 minutes.

**Table 1.** Characteristics of participants in focus groups of 50 asylum seekers and refugees

**ASR characteristics**	***n* (%**)
Sex	Female 24 (48%)Male 26 (52%)
Age range, years	21–68
Length of stay in Wales, range	3 days–6 years
Length of stay in UK, range	1 month–14 years
Countries of origin	
Albania Afghanistan Angola Bangladesh Ethiopia Iran Iraq Libya Malaysia Pakistan Syria Vietnam Zimbabwe	2 (4%)5 (10%)2 (4%)1 (2%)4 (8%)2 (4%)1 (2%)1 (2%)2 (4%)2 (4%)26 (52%)1 (2%)1 (2%)
Spoke English	22 (44%)

ASR = asylum seeker and refugee.

To maintain anonymity, quotations are identified by focus group number, migration status, sex, and place of origin.^33^ Where participants contributed through an interpreter, this interpretation is reported.

The findings are reported under four major themes: navigating the system (Levesque *et al*’s ability to seek and engage); language and communication (ability to reach); matching expectations to NHS resources (ability to pay and to engage); and mental health and trauma (ability to perceive and to seek).^29^

### Navigating the system (ability to seek and engage)

ASRs described a range of specialist staff who facilitated access to services or were able to communicate patients’ needs to health professionals in times of crisis. These included nurses, health visitors, social workers, community support workers funded through Home Office grants, and volunteers. All participants had received help to register with a general practice and most had received support to attend appointments with the GP, dentist, or secondary care, although some participants were unaware of out-of-hours GP services and instead used the emergency department (ED). Participants spoke very positively about this support:

Focus group (FG) 1: *'* *When we were in* [area] *it is a really good system there. … everybody has one centre where they meet and you can discuss all your appointments* [GP and secondary care] *and they follow* *-* *up on everything. Here you have to start afresh on your own* *.'* (Asylum seeker, female, West Africa)

FG 8 (through interpreter): *'* *At night time, the volunteers come if we ask for help* *.'* (Refugee, female, Middle East)

The GP telephone system was an additional barrier. Many used support services or made appointments in person, taking more journeys and time to see a GP:

FG 7:*'* *You ring them and they don’t answer for ages. Sometimes you have to go early morning to the surgery to make an appointment. Or if you try and book an appointment they are giving you an appointment after a week or two weeks. Health* *v* *isitors can help. They can ring and make appointment or talk to the surgery* *.'* (Asylum seeker, male, South Asia)

However, the sustainability of non-statutory support is questionable. In a non-dispersal area, it was found that one NGO was withdrawing support for Syrian refugees after 2 years and this concerned some of the participants, as they were still developing their English-speaking skills. Support from volunteers was tenuous:

FG 8: *'* *There is only probably six to eight of us volunteers left. We started with 12 to 14.* *'* (Female, volunteer)

### Language and communication (ability to reach)

Feeling anxious and unwell exacerbated the language barrier and affected communication during consultations, even for participants with conversation skills.

Participants generally felt reassured when interpretation was available during consultations, either in person or via a telephone language service, to enable them to understand the advice and treatment. However, not all participants rated interpreters highly. Sometimes interpreters spoke a different dialect or could not interpret medical terms fully:

FG 4: *'* *When we go to GP, … a woman is there to interpret for us. … she is not able to interpret properly … she don’t know lots of words* *.'* (Asylum seeker, male, South Asia)

Some participants reported being sent home from the surgery or hospital without treatment, because no interpreters were available. In other cases, practitioners and patients relied on relatives or on Google Translate to interpret consultations, not always accurately:

FG 6 (through interpreter): *'* *I went to see the doctor and he used Google Translate. The doctor thought it was a stroke but it was my tooth* *.'* (Refugee, male, Middle East)

One participant reported clinicians relying on a 12-year-old child’s ability to speak English because her parents could not speak it, leading to them becoming distressed and anxious. The child, a Syrian refugee, had been referred to hospital to repair damage caused by a bullet wound and her consent was sought before undertaking the operation, which health professionals would not ordinarily do for child patients who could speak English:

FG 5 (through interpreter): *'* *I ask for an interpreter but* [hospital] *said your daughter knows English so there is no need. The doctors think my daughter would understand the situation and they took her consent. I made a big mistake, next time I will not allow them to do anything without an interpreter, I have to understand what is going on* *.'* (Refugee, male, Middle East)

There was some mention of the value of written information in languages other than English:

FG 5 (through interpreter): *'* *When my wife was in labour, the hospital provided me with information about induction of labour in Arabic. What is the procedure, what is the side effect* *.'* (Refugee, male, Middle East)

However, many participants said they did not find leaflets helpful, but would prefer information on health on a website — including audio versions — or delivered in person through courses by NGOs and drop-in sessions with health visitors:

FG 1: *'* *It’s good when people can come and talk, like in* [NGO] *‘cause we have a lot of courses there* *.'* (Asylum seeker, female, sub-Saharan Africa)

However, for many, language barriers remained to accessing health promotion and mental health services. There was an awareness that learning to speak English was fundamental to independence, especially accessing care:

FG 8: *'* *The Home Office have said right from the start we’ve got to make them independent. Picking up a phone is much more difficult to make an appointment.* *'* (Volunteer, female)

### Matching expectations to NHS resources (ability to pay and engage)

Although people from lower-income countries generally reported viewing the care they received in Wales positively, there were some misconceptions about the role of the health service; for example, a perception among some that secondary care was only available for seriously ill people. For those participants originating in countries with quite well-developed health systems, there could be frustration at coming across services that were different in Wales, such as pharmacists not prescribing medication. Participants often misunderstood the role of NHS gatekeepers. Some bypassed the GP and went straight to the ED to access specialist care. Others suggested that the service was poor because it was free at the point of care:

FG 6: *'* *In Syria you can go to a private doctor and he will see what the problem is but here you have to wait months* *.'* (Refugee, male, Middle East)

One participant made a positive comparison between the more considered approach to medication in Wales compared with what happened in her home country:

FG 8 (through interpreter): *'* *As Arabs, we like medication for any condition. Medication is like sweets* *…* *here the GP wait and see if problem gets better, that’s good* *.'* (Refugee, female, Middle East)

Participants reported times when access to care was denied, or they felt chronic conditions were mismanaged:

FG 7: *'* *The health service is very good for children. For adults not so good. My husband went to the pharmacy for some pain relief; they did not give him anything. We went to the doctor; the doctor shows a lack of compassion.* *'* (Refugee, female, Middle East)

FG 1: *'* *After two or three weeks the problem happened again and they gave her a course of more tablets. We ask for a specialist to resolve the problem and not just to give tablets. This is not good treatment* *.'* (Asylum seeker, male, Middle East)

One participant reported more extreme challenges associated with engaging with the health service, when a call to NHS 111 was understood as threatening undesired consequences:

FG 6 (through interpreter): *'* *I had a bad experience. I called 111 because my child had injured* [her] *eye so they told me (it was 3am), “If you don’t bring her to the hospital we are going to have to call the police.” So I called the neighbours to take me to hospital* *.'* (Refugee, female, Middle East)

Other challenges to engaging with health care were very particular to the ASR group. Dispersal of ASs disrupted continuity of care:

FG 4: *'* *When* [son aged 5] *was in England, he was seeing* [mental health care]*, so when I come here we told them many times* [he needs mental health care]*, but they didn’t do anything. I have shown* [GP] *lots of pictures of* [son] *self-harming, but they will not respond* *.'* (Asylum seeker, male, South Asia)

Dissatisfied participants were unaware that they could change their GP or dentist; ASs believed they needed permission from NASS to change provider:

FG 4: *'* *I wanted to change my GP but I haven’t got any letter from Home Office* *.'* (Asylum seeker, female, South Asia)

The cost of proprietary medicines, like infant paracetamol, was a challenge for ASs. Although patients’ travel costs to health care can be reclaimed, awareness of the process was low, and family members were not covered:

FG 1 [through interpreter]: *'* *I don’t even have enough money to eat and provide essential things for my family, and I have to spend most of this money on travel going in and out of the hospital* *.'* (Asylum seeker, male, Middle East)

One refused AS (without public funds) believed he had no free access to see the GP or dentist, and relied on donations from parishioners to cover the £60 cost of dental treatment. Participants understood that they were responsible for their health, and health care was for illnesses. They described remedies for treating minor ailments such as herbs and vinegar to treat colds and fever. They avoided going out in bad weather to prevent ill health. Participants mentioned the importance of eating fresh food and taking exercise, but wanted support to do this well. They mentioned volunteering, sport, and keeping busy as ways to build resilience against mental health problems:

FG 2: *'* *You need to look after yourself because you cannot easily get access to the GP so you have to make sure that you stay healthy* *.'* (Refugee, male, Middle East)

### Mental health and trauma (ability to perceive and seek)

Participants in this study were happy to talk about mental health, although very few talked about their sanctuary-seeking journeys. Instead, they focused on stress caused by isolation and uncertainty. One older participant had lost his first wife in Syria. Now they were living in Wales on a low income, their second wife had been diagnosed with cancer, and their children and grandchildren were still in refugee camps in Jordan. They were not the only participant to report feeling helpless:

FG 8 (through interpreter): *'* *I like to be in big city. I want to be in contact with Arabic young people because it is difficult to express myself in English. Transportation is difficult here and* [I’m] *very lonely* *.'* (Refugee, female, Middle East)

FG 5 (through interpreter): *'...* *the experience that I have is killing me every day* *.'* (Refugee, male, Middle East)

Participants generally talked about feeling sad rather than feeling mentally unwell. They acknowledged mental wellbeing as key to staying healthy, but struggled with the weather and the perceived isolation associated with modern technology. However, some refugees believed, once physiological and safety needs were met, they should forget the past, concentrate on the present, and trust their god to prevent mental ill health:

FG 7 (through interpreter): *'* *We leave our problems behind and not remember the past, and be grateful for what we have. As Arab people we do not complain about mental health. Our religion helps us to overcome all these problems* *.'* (Refugee, female Middle East)

Some participants mentioned that they would not talk to their GP or other health professionals about mental health, fearing it would instigate prescribed medication, which they believed would be detrimental to long-term health and make matters worse. They preferred a psychosocial response to mental health care:

FG 7: *'* *I would not go to the GP with mental problems. Because it will cause more depression* *...* *because they will prescribe tablets* *.'* (Asylum seeker, female, East European)

*FG 7: 'If my son was sad, I would talk to him, show more kindness and give him hugs*.' (Refugee, male, South East Asia)

While some recognised that health care might help, they found access to mental health care a challenge, experiencing long waits or no care at all:

FG 2: *'* *I think it’s very hard to get mental health support here … from my experience* [seeking mental health support for teenage daughter]*.'* (Asylum seeker, male, South East Asia)

Participants reported that mental health services did not generally offer counselling or therapy through interpreters. Participants who had received counselling reported mixed feelings:

FG 1: *'* *I don’t think I need more counselling. I felt it helped me, but sometimes you don’t want to talk about stuff, and when you do talk, it is more distressing because … you are going back to it.* *'* (Asylum seeker, female, Central Africa)

## Discussion

### Summary

This is the first study to explore the views of ASRs across Wales about health care. In terms of navigating the system, most understood the role of general practice in providing and coordinating care, but were unaware of some services, such as out of hours.^21^ They valued the role of specialist NHS-funded services and grant-aided NGOs in facilitating access to health care. Difficulties with language and communication featured strongly as barriers to health care, alongside health literacy, unrecognised needs, and the cost of travel to appointments. Written information about health care is limited in accessibility to those with language needs, and few participants were accessing preventive health services.^34^ Typically, ASRs framed their responses through the perspective of their home country, leading to some mismatch between expectations and what they experienced.^23^ Participants recognised the importance of mental health, but many did not see it as something for which they would seek support from a GP. Others expressed disappointment at the state of mental health care.

### Strengths and limitations

The findings cannot be generalised to all ASRs. Barriers to access were probably underestimated, as service users were more likely to participate; however, the researchers judge that their use of peer researchers to recruit participants across Wales ameliorated this potential bias. It is recognised that focus groups may have inhibited some views, however hypothetical health scenarios were used to ease people into discussions and it was found most responded to these situations as they were not directly referencing their personal experience. Many participants were not able to communicate their views in English, and although interpreters were provided and the researchers ensured that short sentences were used, it was not always possible to use a professional interpreter.

### Comparison with existing literature

Understanding user perspectives is vital when exploring ways to improve the delivery of health care.^35^ Levesque *et al*’s conceptual framework of access to health care was found to be a useful framework for organising the data, and for identifying the barriers and enablers faced by ASRs. Although the model does not include migration as one of the determinants operationalising access to health care, it functioned well in examining the experience of ASRs. The results are consistent with previous findings that:

- many participants used NGOs for support;^3,36^
- although refugees had housing and income, many suffered post-resettlement stress;^37^
- unmet expectations generated mistrust of GPs, resulting in repeat visits or ED attendances;^1,38^
- weak interpretation services weakens consultations;^2,18,39^
- and interpreters cannot interpret all medical terms.^2,40^

It was found that ASRs’ first point of contact with health services with frontline staff, such as GP receptionists, emergency paramedics, and pharmacists, was often weakened by a lack of effective interpretation.

Most ASRs openly discussed mental health and wellbeing, in contrast to previous work.^41^ They confirmed that their migration journey had contributed to poor health.^42^ Nevertheless, recent migrants preferred to forget the past once they were safe in their host country. Unexpectedly, some ASRs were wary of consulting GPs for mental health; less surprisingly, language barriers deterred others from accessing mental health care.^43^ Fortunately several ASRs recognised that good mental health enhances coping strategies when integrating into new communities.^44,45^

### Implications for practice

Since the 1951 Refugee Convention,^5^ the UNHCR has promoted the right of refugees everywhere to have the same access to health care as the host population. The Welsh Government has sought to operationalise this principle in its 2019 plan for a Nation of Sanctuary for refugees and ASs,^14^ which includes a requirement for all ASRs to have access to health services (including mental health services) throughout their sanctuary-seeking journey. The authors' research suggests the implementation of a multi-agency systems approach to achieving this goal. To facilitate patient-centred care, not only during GP consultations but also in interactions with other practice staff, access to competent interpretation is crucial. To improve experience of care, manage expectations, and reduce distrust, the authors recommended continuity of care,^46^ flexibility in the appointment system,^3,22^ and education about entitlements targeted at both ASRs and health providers at all levels, in particular the legal entitlement to interpretation services.

ASRs need continued investment in support services including specialist health professionals and NGOs. Volunteers provide patient-centred care that tailors consultations to need. Such support should continue until ASRs are familiar with services and achieve basic English language proficiency. The added cognitive load experienced by patients trying to speak in English during consultations can inhibit understanding and communication.^47^ ASRs recognised that these services had enabled them to navigate an unfamiliar system of care and were there to advocate on their behalf.^2^ As such, mainstream healthcare providers need to work closely with these support services who understand how best to respond to ASRs. They should also offer access to interpreters in situations where mental health care is sought rather than waiting for people to become proficient in English before providing care, as it may take some ASRs a number of years to reach language proficiency.^48^

Finally, commissioners and service providers should encourage innovative ways of caring for refugees and asylum seekers; and evaluate any such innovation rigorously. This is especially pertinent at this time when remote GP consulting is increasingly being used owing to the COVID-19 pandemic. This is an added layer of complexity and the impact of such changes on ASRs needs to be fully evaluated. There is potential risk that remote GP consulting may increase health inequality by reducing access to interpreters with the added barrier of digital poverty, which can prevent online access in the first place.

## References

[1] Rafighi E, Poduval S, Legido-Quigley H, Howard N (2016). National Health Service principles as experienced by vulnerable London migrants in "austerity Britain": a qualitative study of rights, entitlements, and civil-society advocacy. Int J Health Policy Manag.

[2] O'Donnell CA, Higgins M, Chauhan R, Mullen K (2007). "They think we're OK and we know we're not". A qualitative study of asylum seekers' access, knowledge and views to health care in the UK. BMC Health Serv Res.

[3] Fang ML, Sixsmith J, Lawthom R (2015). Experiencing 'pathologized presence and normalized absence'; understanding health related experiences and access to health care among Iraqi and Somali asylum seekers, refugees and persons without legal status. BMC Public Health.

[4] Home Office (2021). Asylum. Point of claim information booklet. https://assets.publishing.service.gov.uk/government/uploads/system/uploads/attachment_data/file/979565/Asylum_Point_of_Claim_Booklet_-_April_2021.pdf.

[5] United Nations (1951). Convention relating to the status of refugees. https://www.ohchr.org/en/professionalinterest/pages/statusofrefugees.aspx.

[6] United Nations Declaration of Human Rights (1948). Adopted by the United Nations General Assembly. Geneva: UNHCR. http://www.unhcr.ch.

[7] United Nations High Commissioner for Refugees (1981). Protection of asylum seekers in situations of large-scale influx No. 22 (XXXII). https://www.unhcr.org/uk/%20excom/exconc/3ae68c6e10/protection-asylum-seekers-situations-large-scale-influx.html.

[8] Milosevic D, Cheng I-H, Smith MM (2012). The NSW Refugee Health Service — improving refugee access to primary care. Aust Fam Physician.

[9] Hunter P (2016). The refugee crisis challenges national health care systems: countries accepting large numbers of refugees are struggling to meet their health care needs, which range from infectious to chronic diseases to mental illnesses. EMBO Rep.

[10] Pavli A, Maltezou H (2017). Health problems of newly arrived migrants and refugees in Europe. J Travel Med.

[11] United Nations High Commission of Refugees (2019). Global trends: forced displacement in 2018. https://www.unhcr.org/uk/statistics/unhcrstats/5d08d7ee7/unhcr-global-trends-2018.html.

[12] World Health Organization Migration and health: key issues. https://www.euro.who.int/en/health-topics/health-determinants/migration-and-health/migration-and-health-in-the-european-region/migration-and-health-key-issues.

[13] United Nations International Children’s Emergency Fund Refugee and migrant crisis in Europe: is health care accessible?. https://www.unicef.org/eca/sites/unicef.org.eca/files/UNICEF%20Advocacy%20Brief%20Health.pdf.

[14] Welsh Government (2019). Nation of sanctuary — refugee and asylum seeker plan. https://gov.wales/sites/default/files/publications/2019-03/nation-of-sanctuary-refugee-and-asylum-seeker-plan_0.pdf.

[15] Welsh Equality, Local Government and Communities Committee (2017). “I used to be someone.” Refugees and asylum seekers in Wales.

[16] Home Office (2020). Immigration statistics data tables: year ending December 2019. https://www.gov.uk/government/statistics/immigration-statistics-year-ending-december-2019.

[17] Home Office Asylum accommodation and support: schedule 2. Statement of requirements. https://www.emcouncils.gov.uk/write/Asylum_Accommodation_and_Support_Statement_of_requirements.pdf.

[18] Bradby H, Humphris R, Newall D (2015). Public health aspects of migrant health: a review of the evidence on health status for refugees and asylum seekers in the European Region.

[19] Renton Z, Hamblin E, Clements K (2016). Delivering the healthy child programme for young refugee and migrant children.

[20] Chiarenza A, Dauvrin M, Chiesa V (2019). Supporting access to healthcare for refugees and migrants in European countries under particular migratory pressure. BMC Health Serv Res.

[21] Kang C, Tomkow L, Farrington R (2019). Access to primary health care for asylum seekers and refugees: a qualitative study of service user experiences in the UK. Br J Gen Pract.

[22] Bunting R (2009). Asylum seeker and refugee health needs assessment.

[23] O'Donnell CA, Higgins M, Chauhan R, Mullen K (2008). Asylum seekers' expectations of and trust in general practice: a qualitative study. Br J Gen Pract.

[24] Welsh Government (2015). Wellbeing of Future Generations (Wales) Act 2015.

[25] Alvarez AR, Gutiérrez LM (2001). Choosing to do participatory research. J Community Pract.

[26] Fête M, Aho J, Benoit M (2019). Barriers and recruitment strategies for precarious status migrants in Montreal, Canada. BMC Med Res Methodol.

[27] George S, Duran N, Norris K (2014). A systematic review of barriers and facilitators to minority research participation among African Americans, Latinos, Asian Americans, and Pacific Islanders. Am J Public Health.

[28] Khanom A, Alanazy W, Ellis L (2019). The health experiences of asylum seekers and refugees: technical report of the HEAR study.

[29] Levesque J-F, Harris MF, Russell G (2013). Patient-centred access to health care: conceptualising access at the interface of health systems and populations. Int J Equity Health.

[30] Staniszewska S, Brett J, Simera I (2017). GRIPP2 reporting checklists: tools to improve reporting of patient and public involvement in research. BMJ.

[31] Ritchie J, Spencer L, Bryman A, Burgess R. G (1994). Analysing Qualitative Data.

[32] Green J, Thorogood N (2014). Qualitative Methods for Health Research.

[33] Bowling A, Ebrahim S (2005). Handbook of Health Research Methods: Investigation, Measurement and Analysis.

[34] Aspinall PJ (2014). *Vulnerable migrants, gypsies and travellers, people who are homeless, and sex workers: a review and synthesis of interventions and service models that improve access to primary care and reduce risk of avoidable admission to hospital.* University of Kent, Canterbury. www.gov.uk/government/uploads/system/uploads/attachment_data/file/305912/Inclusive_Practice.pdf.

[35] Crawford MJ, Rutter D, Manley C (2002). Systematic review of involving patients in the planning and development of health care. BMJ.

[36] El-Gamal S, Hanefeld J (2020). Access to health-care policies for refugees and asylum-seekers. Int J Migr Health Soc Care.

[37] O'Donnell AW, Stuart J, O'Donnell KJ (2020). The long-term financial and psychological resettlement outcomes of pre-migration trauma and post-settlement difficulties in resettled refugees. Soc Sci Med.

[38] Birkhäuer J, Gaab J, Kossowsky J (2017). Trust in the health care professional and health outcome: a meta-analysis. PLoS One.

[39] Bhatia R, Wallace P (2007). Experiences of refugees and asylum seekers in general practice: a qualitative study. BMC Fam Pract.

[40] Crawshaw A, Hornigold R, Mandal S, Campos-Matos I (2019). Caring for your migrant patients and providing for their needs. Practice Nursing.

[41] Shannon PJ, Wieling E, Simmelink-McCleary J, Becher E (2015). Beyond stigma: barriers to discussing mental health in refugee populations. J Loss Trauma.

[42] World Health Organization Regional Office for Europe (2018). Mental health promotion and mental health care in refugees and migrants. Technical guidance.

[43] Wong EC, Marshall GN, Schell TL (2006). Barriers to mental health care utilization for U.S. Cambodian refugees. J Consult Clin Psychol.

[44] Papadopoulos I, Lees S, Lay M, Gebrehiwot A (2004). Ethiopian refugees in the UK: migration, adaptation and settlement experiences and their relevance to health. Ethn Health.

[45] Ozbay F, Johnson DC, Dimoulas E (2007). Social support and resilience to stress: from neurobiology to clinical practice. Psychiatry.

[46] Burchill J, Pevalin D (2012). Barriers to effective practice for health visitors working with asylum seekers and refugees. Community Pract.

[47] Chen IJ, Chang CC (2009). Cognitive load theory: an empirical study of anxiety and task performance in language learning. Electronic Journal of Research in Educational Psychology.

[48] Morrice L, Tip LK, Collyer M, Brown R (2021). ‘You can’t have a good integration when you don’t have a good communication’: English-language learning among resettled refugees in England. J Refug Stud.

